# Supplementary material for: Validation of body surface colonic mapping (BSCM) against high resolution colonic manometry for evaluation of colonic motility
Source: Sci Rep. 2024 Feb 28;14:4842. doi: 10.1038/s41598-024-54429-7 (PMC10902299; doi:10.1038/s41598-024-54429-7)
Supplement: Supplementary file 1 — Supplementary Information. [file 41598_2024_54429_MOESM1_ESM.docx]

**Validation of body surface colonic mapping against high resolution colonic manometry in evaluation of colonic motility**

Sean HB Seo, Cameron I Wells, Tully Dickson, David Rowbotham, Armen Gharibans, Stefan Calder, Ian Bissett, Greg O’Grady and Jonathan C Erickson

# TABLE OF CONTENTS

| **Table S1:** | Frequency proportions - intrinsic vs. sequential frequency | Page 2 |
| --- | --- | --- |
| **Figure S1:** | Spatial analysis: All subjects; major phases | Page 4 |
| **Figure S2:** | Spatial analysis: All subjects; 10 minute epochs | Page 8 |
| **Figure S3:** | Analysis of HRCM frequency distributions pre- and post-meal | Page 12 |
|  |  |  |

# Table S1: CMP activity proportions per frequency bandwidth - intrinsic vs. sequential frequency.

|  | **Intrinsic frequency** | | **Sequential frequency** | |
| --- | --- | --- | --- | --- |
|  | **2-4 cpm** | **4-10 cpm** | **2-4 cpm** | **4-10 cpm** |
| **Subject 1** | 48.27 | 34.52 | 20.21 | 49.20 |
| **Subject 2** | 54.65 | 23.68 | 36.18 | 42.76 |
| **Subject 3** | 47.92 | 34.30 | 34.72 | 49.46 |
| **Subject 4** | 39.00 | 43.09 | 14.23 | 55.55 |
| **Subject 5** | 51.71 | 24.03 | 31.48 | 42.43 |
| **Subject 6** | 44.85 | 34.58 | 33.71 | 42.70 |
| **Subject 7** | 38.47 | 38.52 | 14.74 | 47.14 |
| Mean | 46.41 | 33.25 | 26.47 | 47.03 |
| Std | 6.08 | 7.14 | 9.72 | 4.85 |
| Median | 47.92 | 34.52 | 31.48 | 47.14 |

**
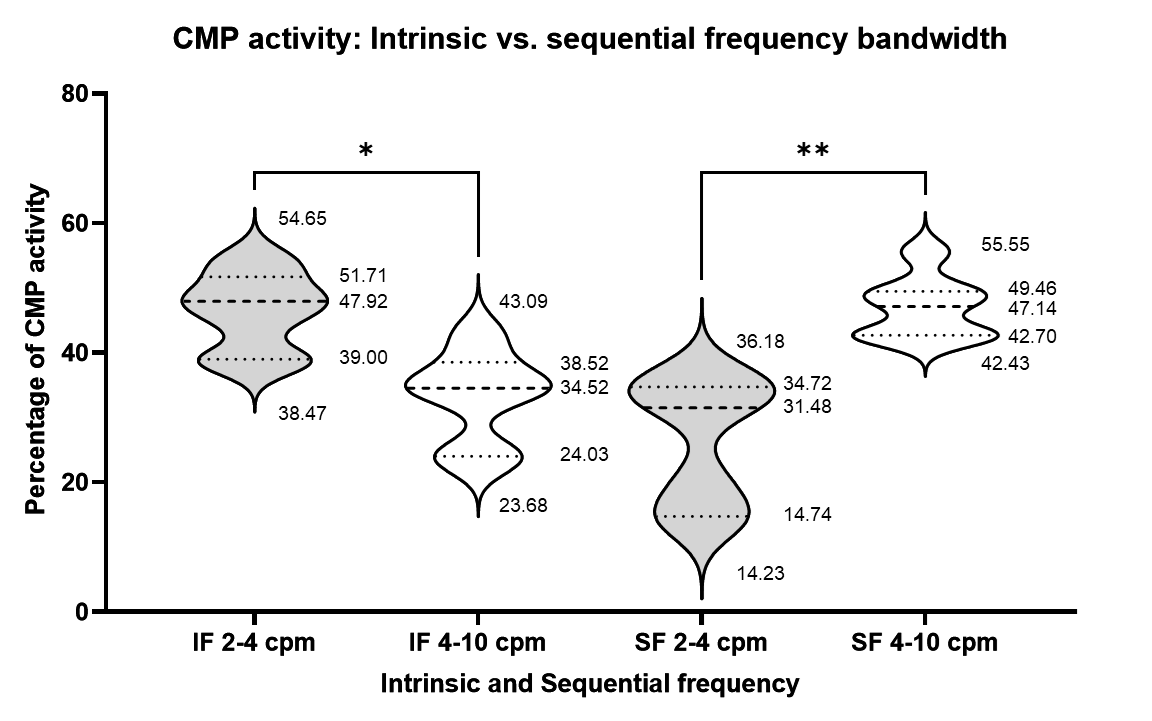
Table S1: CMP activity proportions per frequency bandwidth - intrinsic vs. sequential frequency:** Frequency was analyzed in two ways from the manometry data. *Intrinsic frequency* is the rate of CMP activity detected on each manometer independent of other sensor data, thus representing the actual physiological frequency of the CMPs. *Sequential frequency* is the frequency of all multifocal CMP activities combined; i.e. all active colonic segments are viewed as one single active organ (when only one region is active, the frequency will equal intrinsic frequency). Intrinsic frequency of the colon is predominantly around 2-4 cpm, however, the proportions of CMP activity seen via the sequential frequency bandwidths are the opposite and the 4-10 cpm range becomes the dominant bandwidth. The sum of the two bandwidth values do not add up to 100 as the proportions of minor frequency bandwidths below 2 cpm and above 10 cpm are not shown.

# Figure S2. Spatial analysis: All subjects; major phases

Subject 1


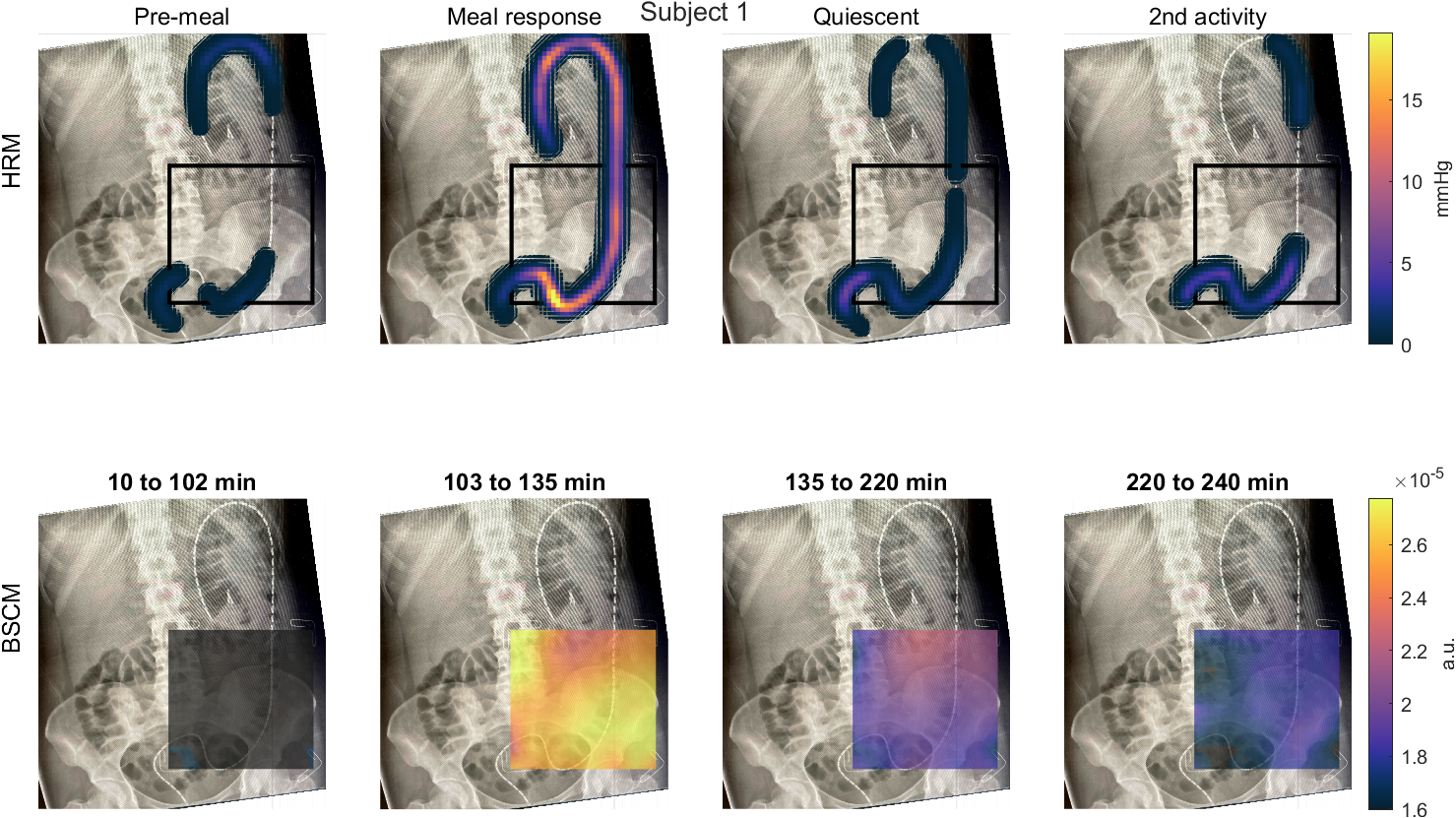


Subject 2


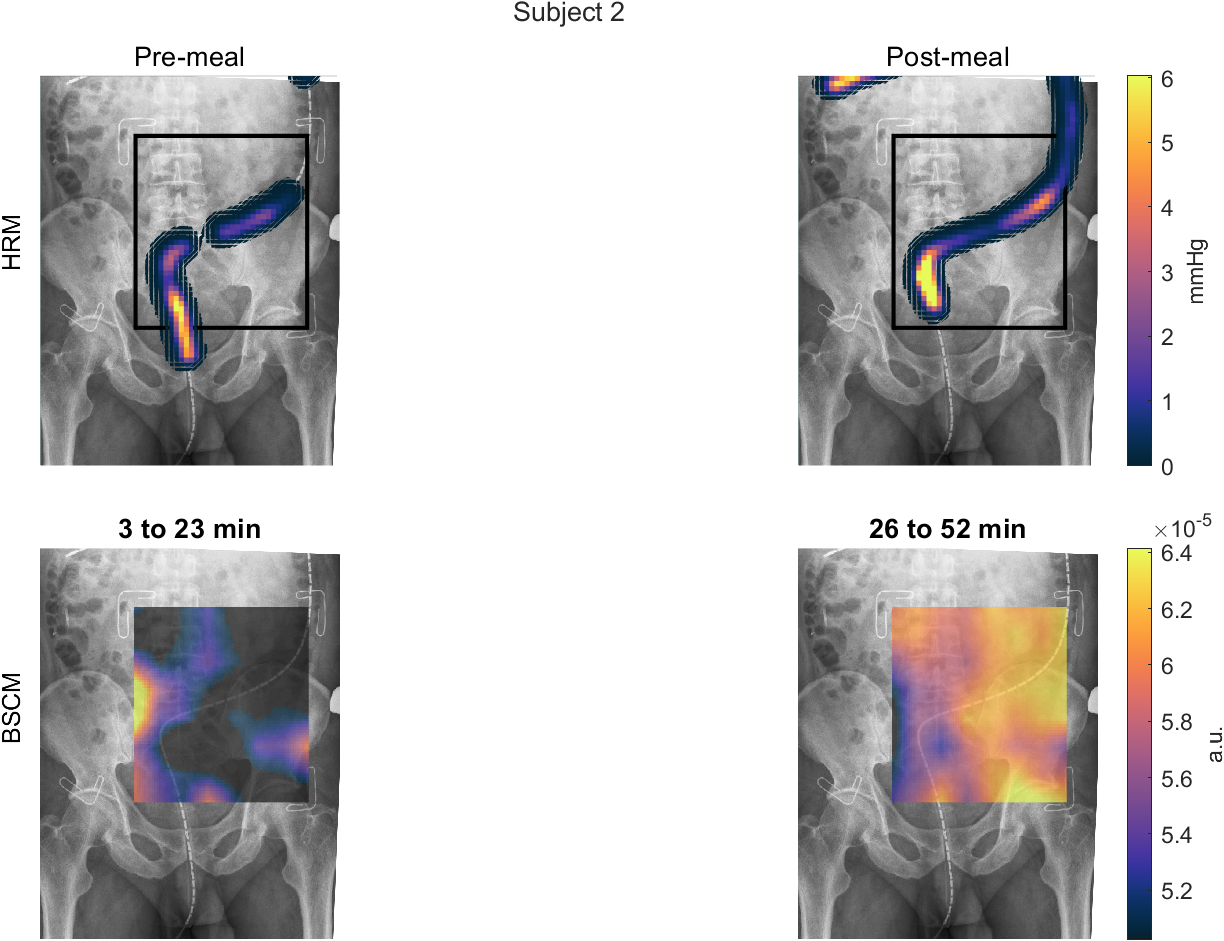


Subject 3
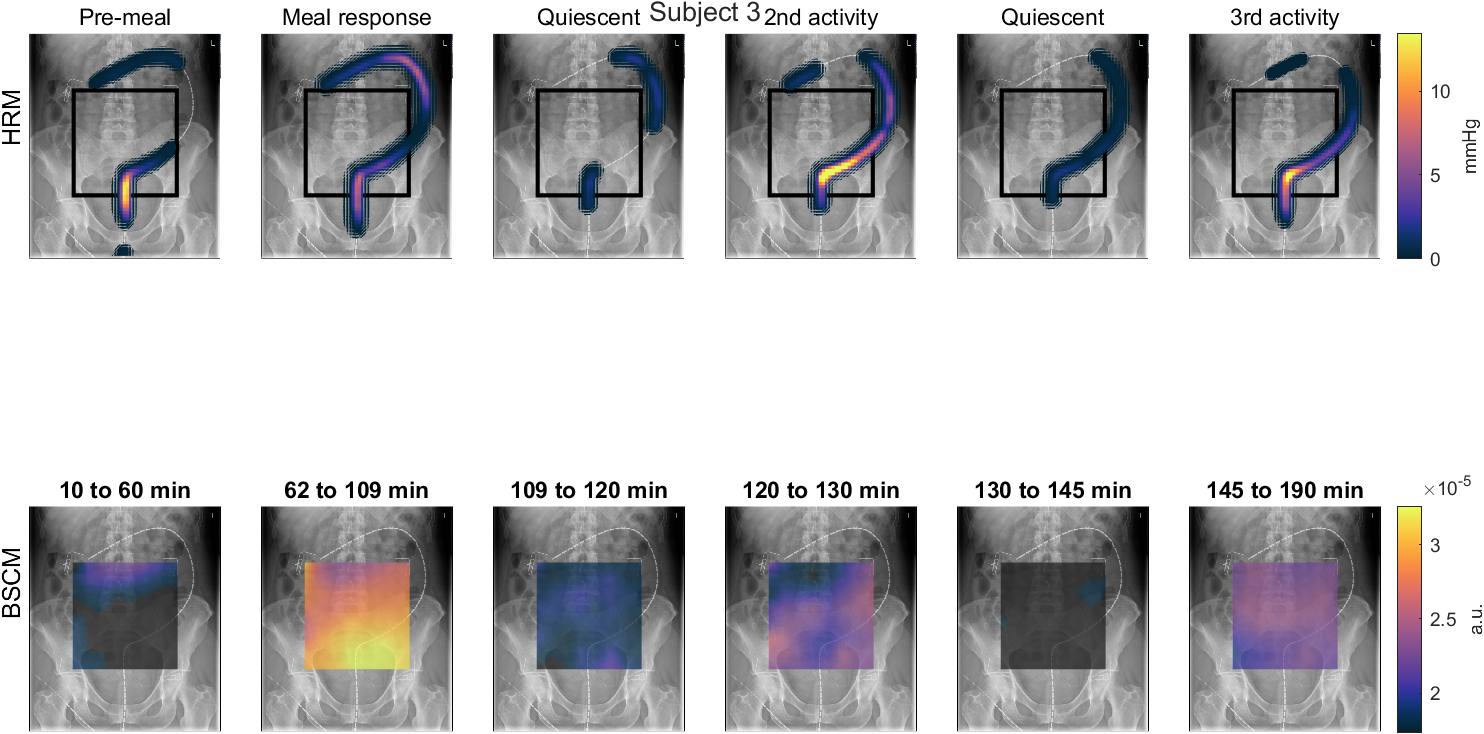


Subject 4


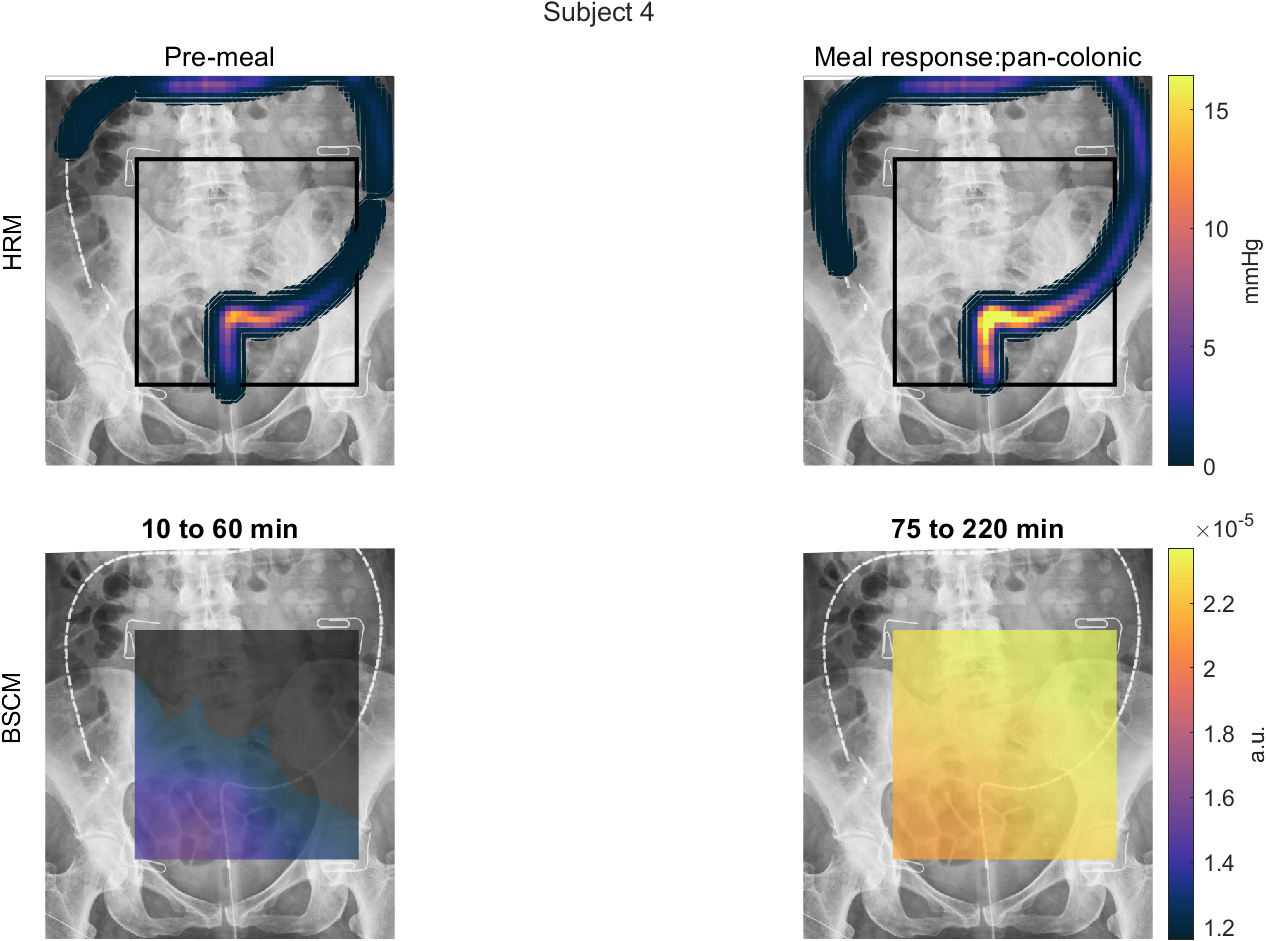


Subject 5
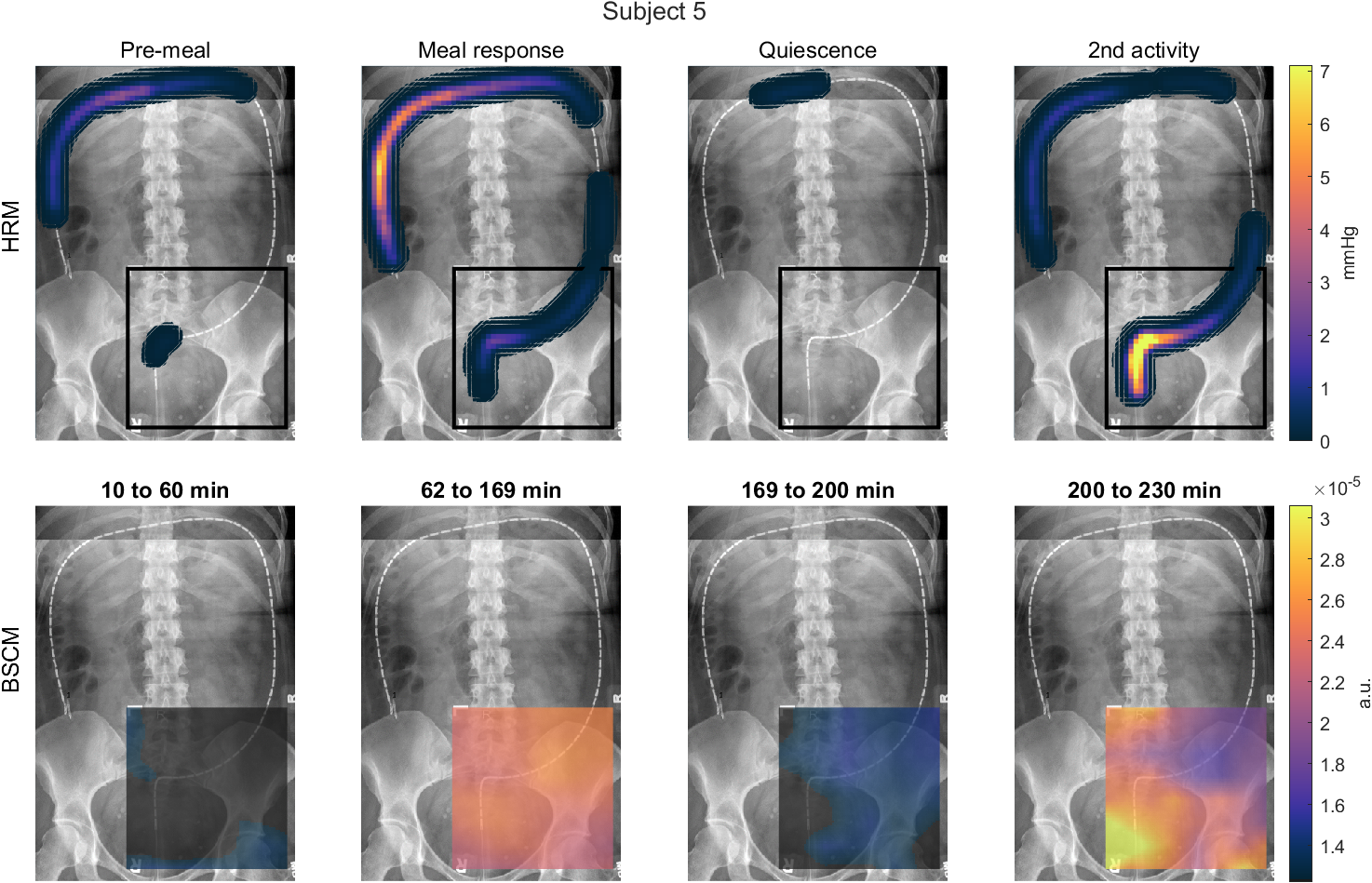


Subject 6


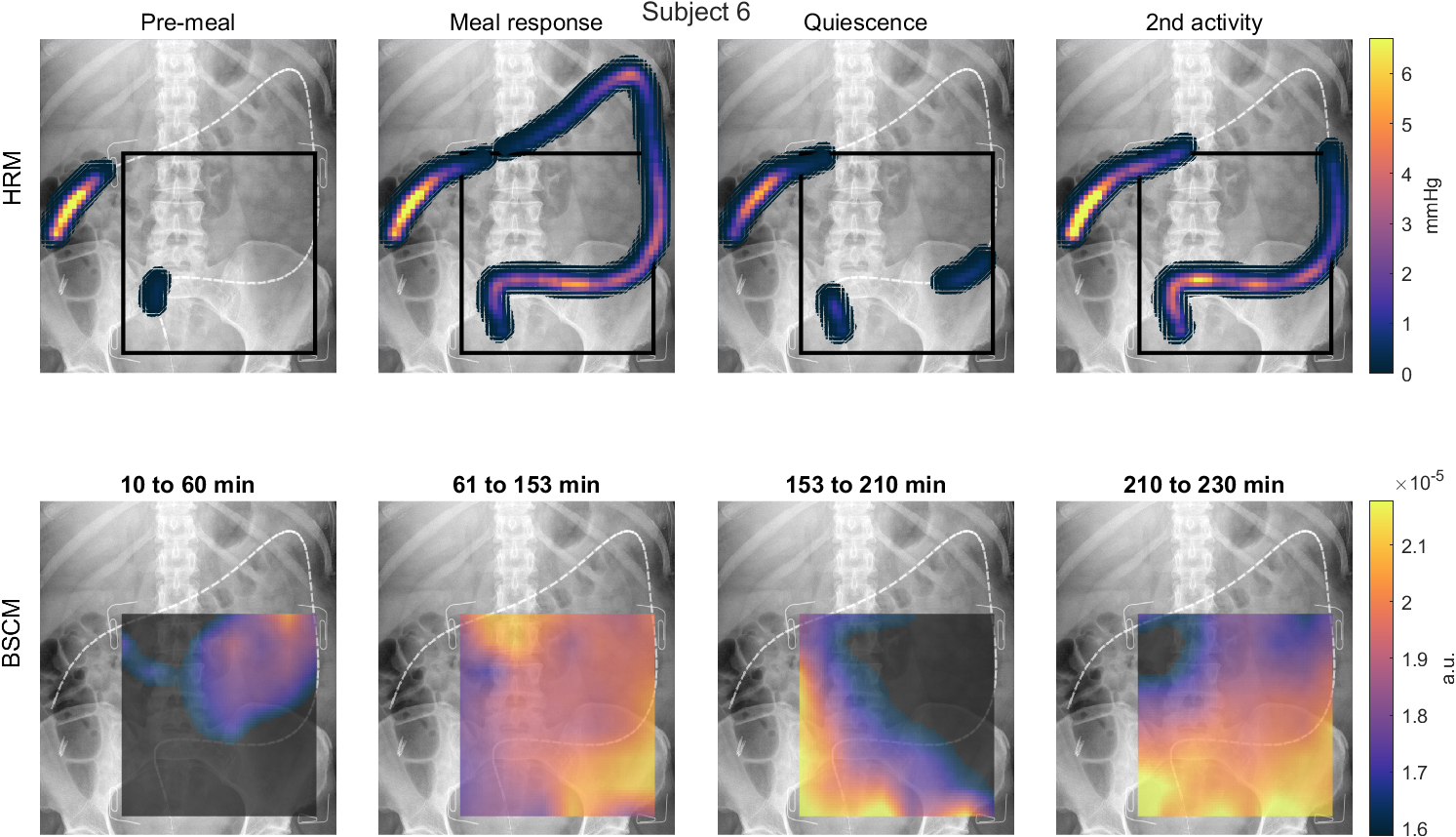


Subject 7


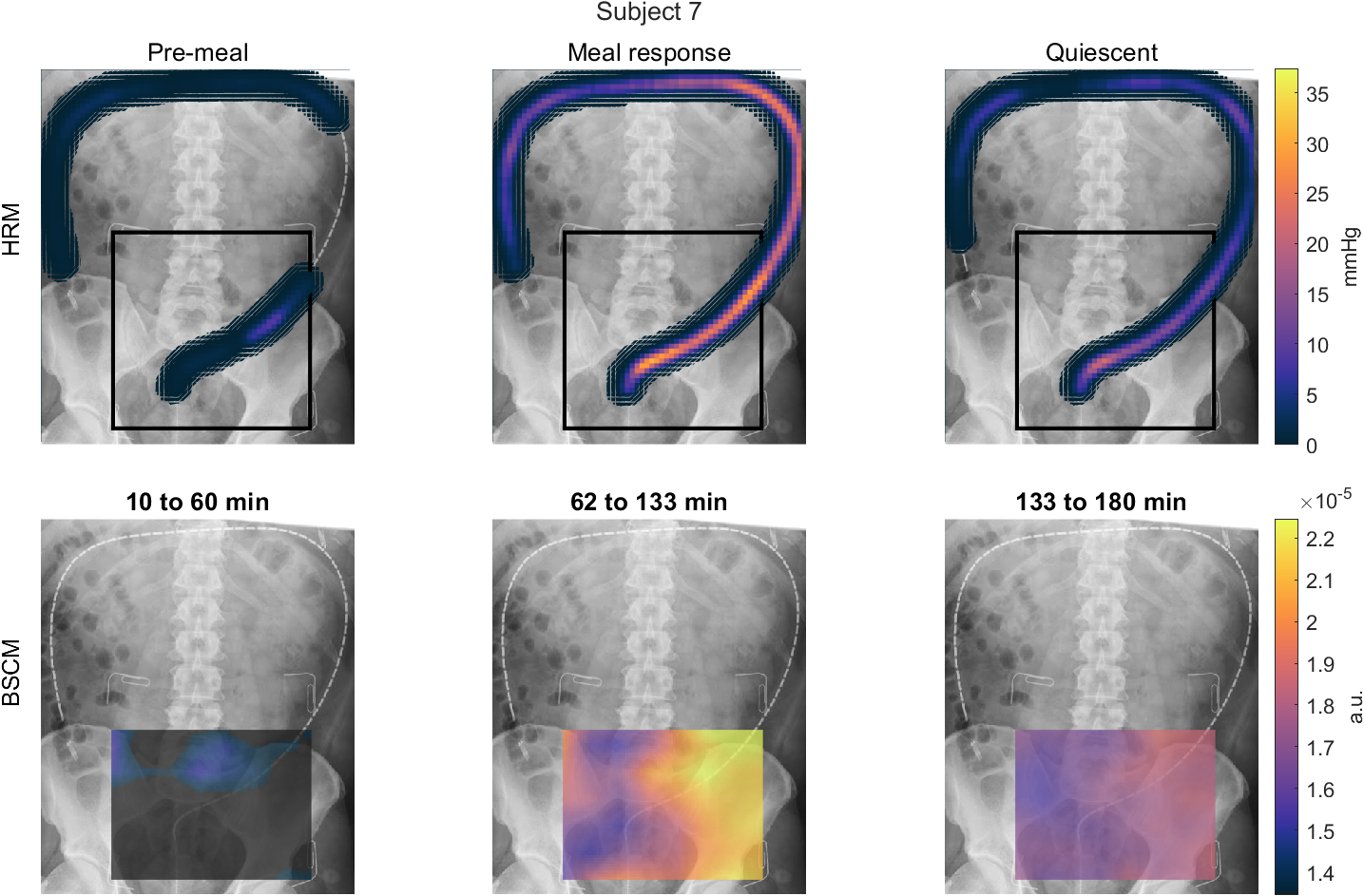


# Figure S1. Spatial analysis: All subjects; 10 minute epochs.

Subject 1


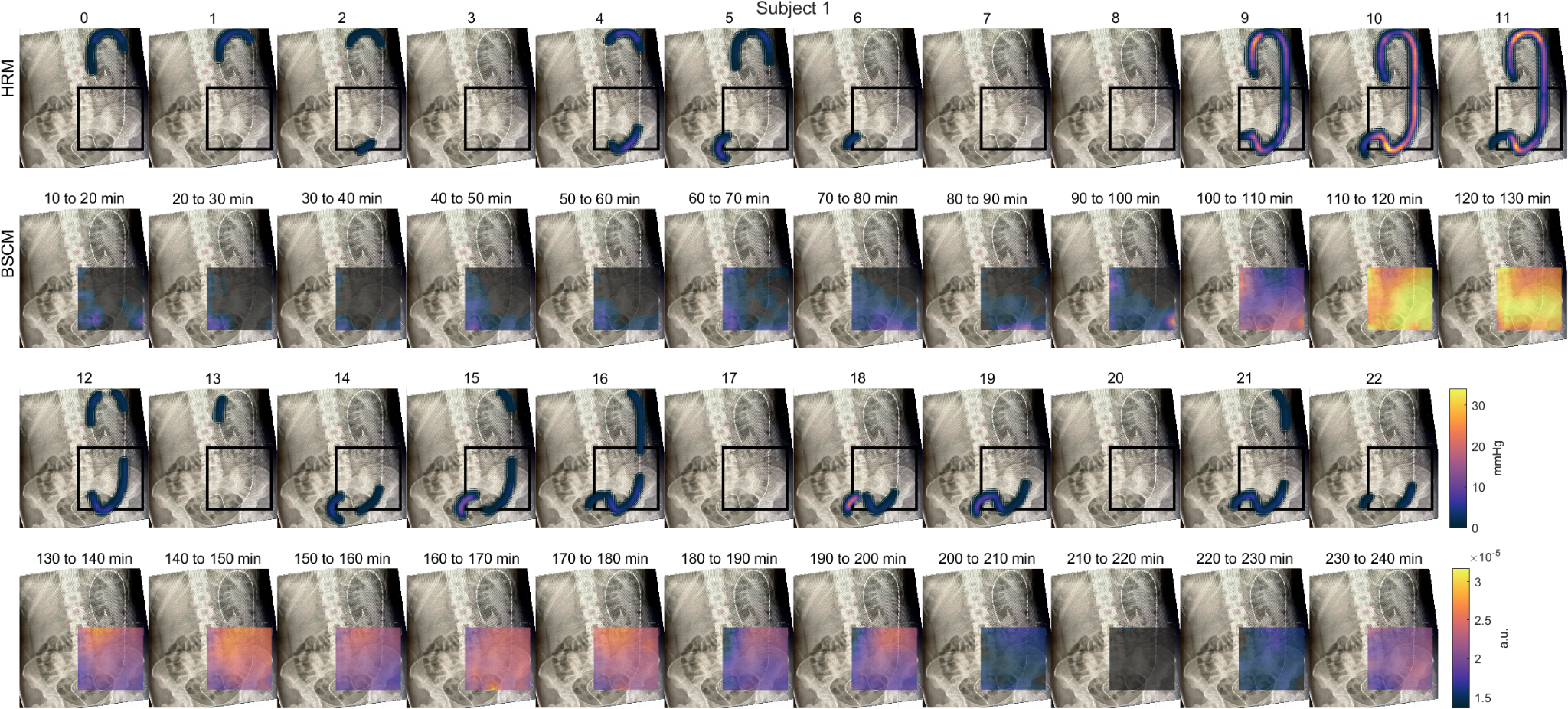


Subject 2
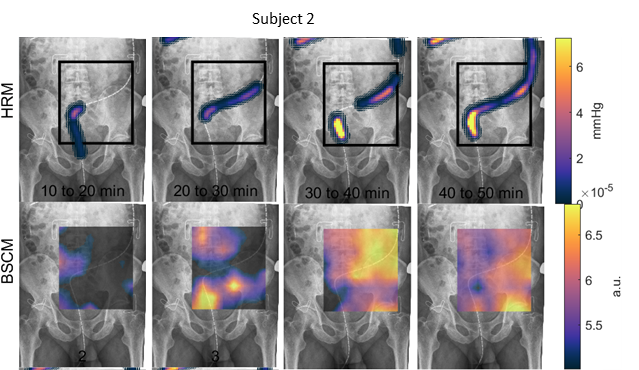


Subject 3
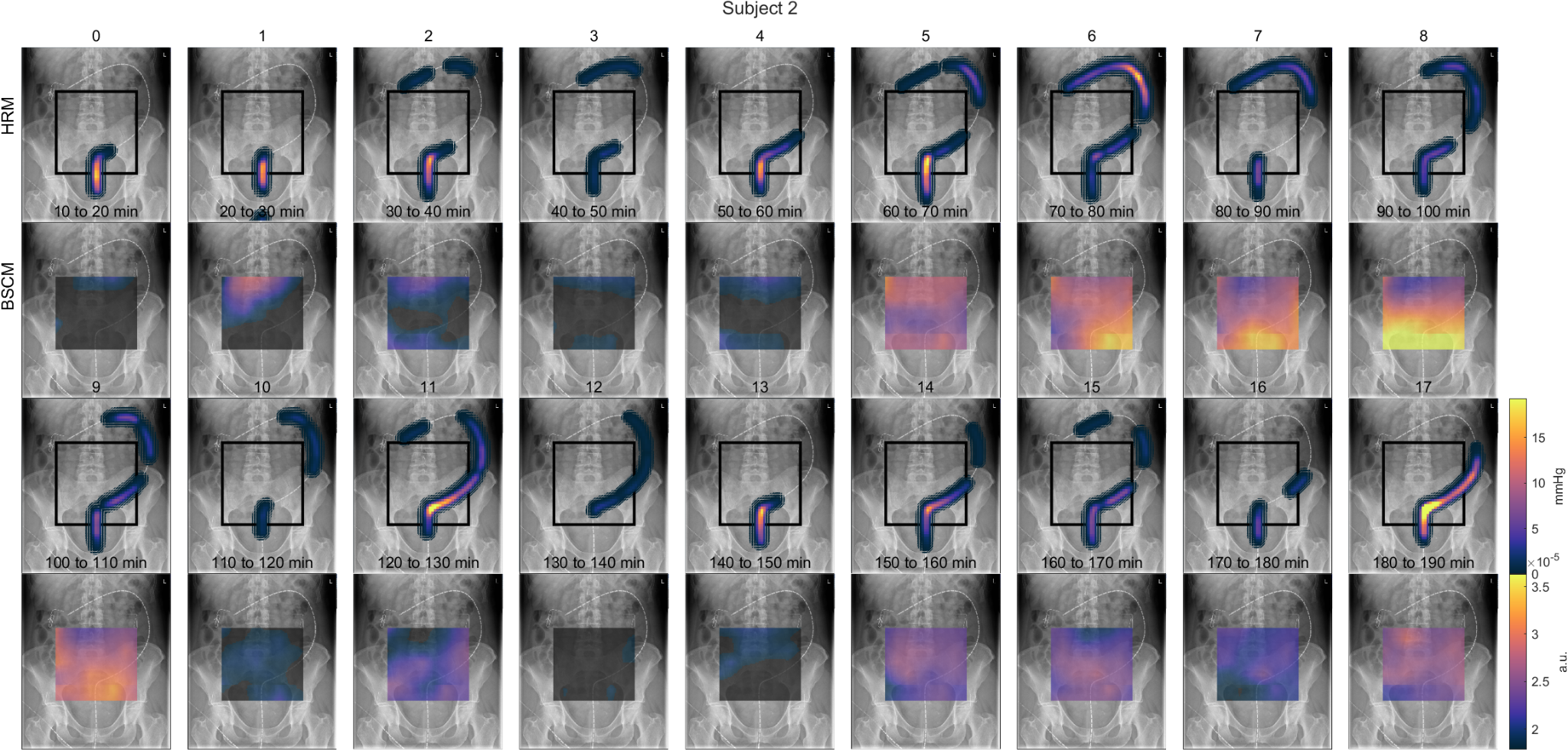


Subject 4
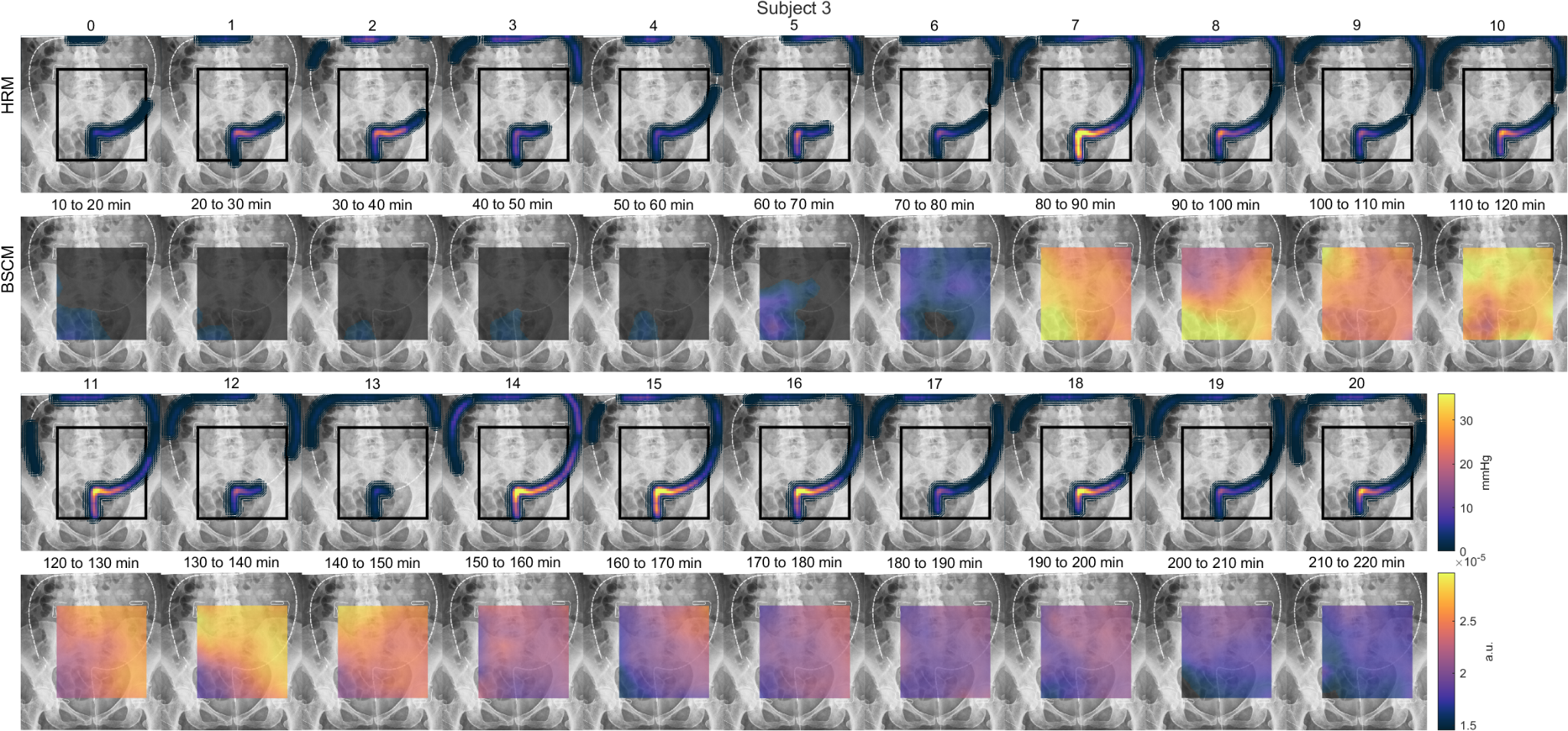


Subject 5
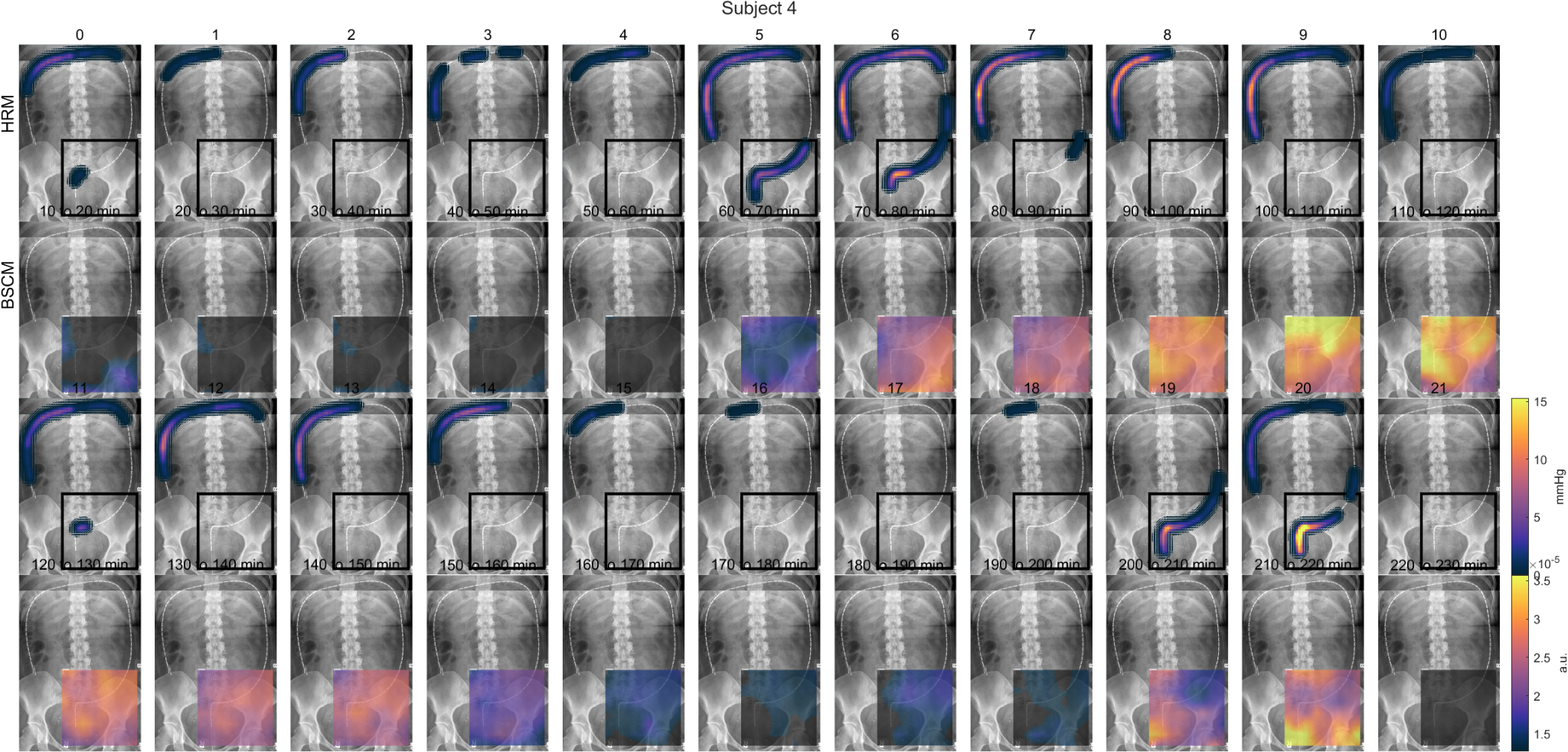


Subject 6
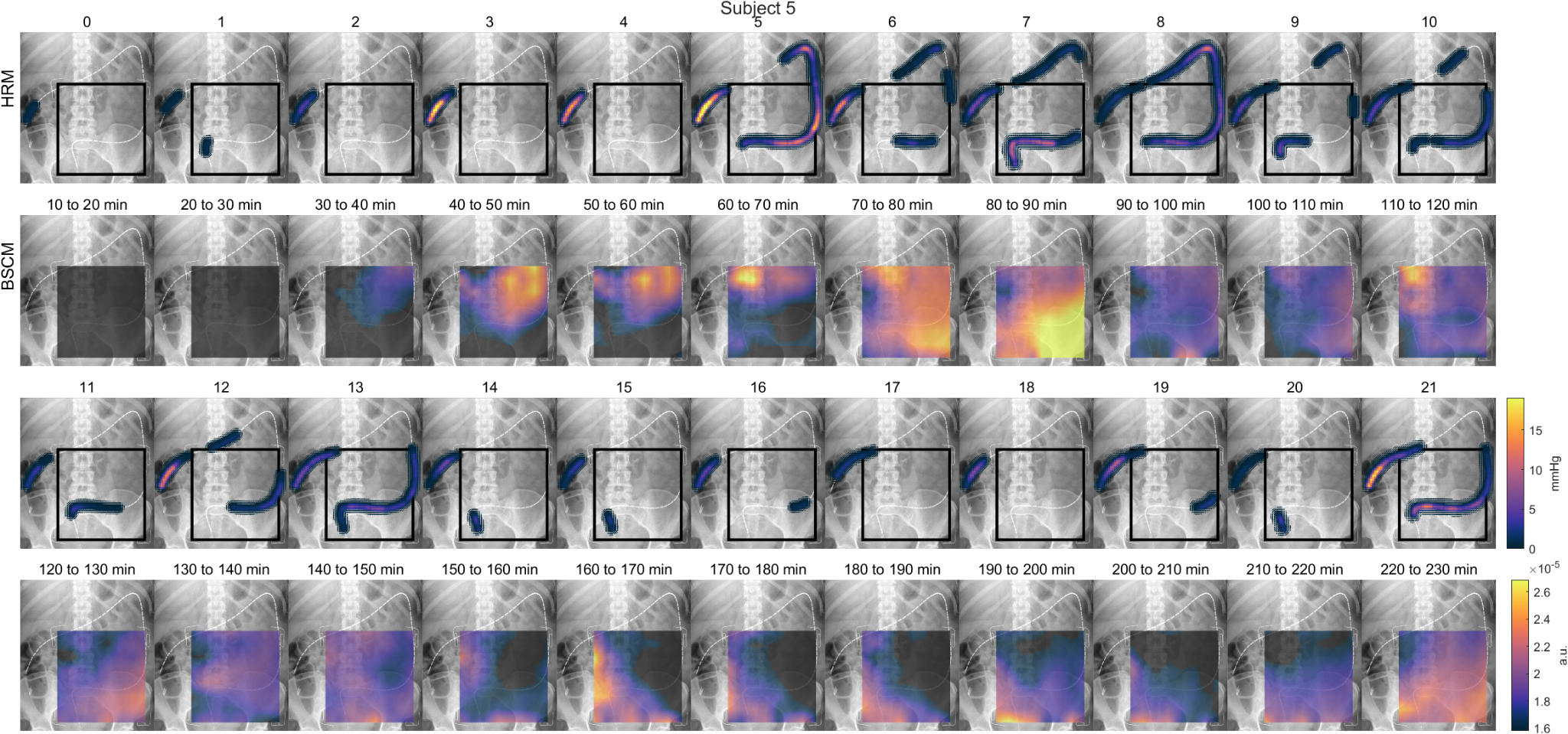


Subject 7
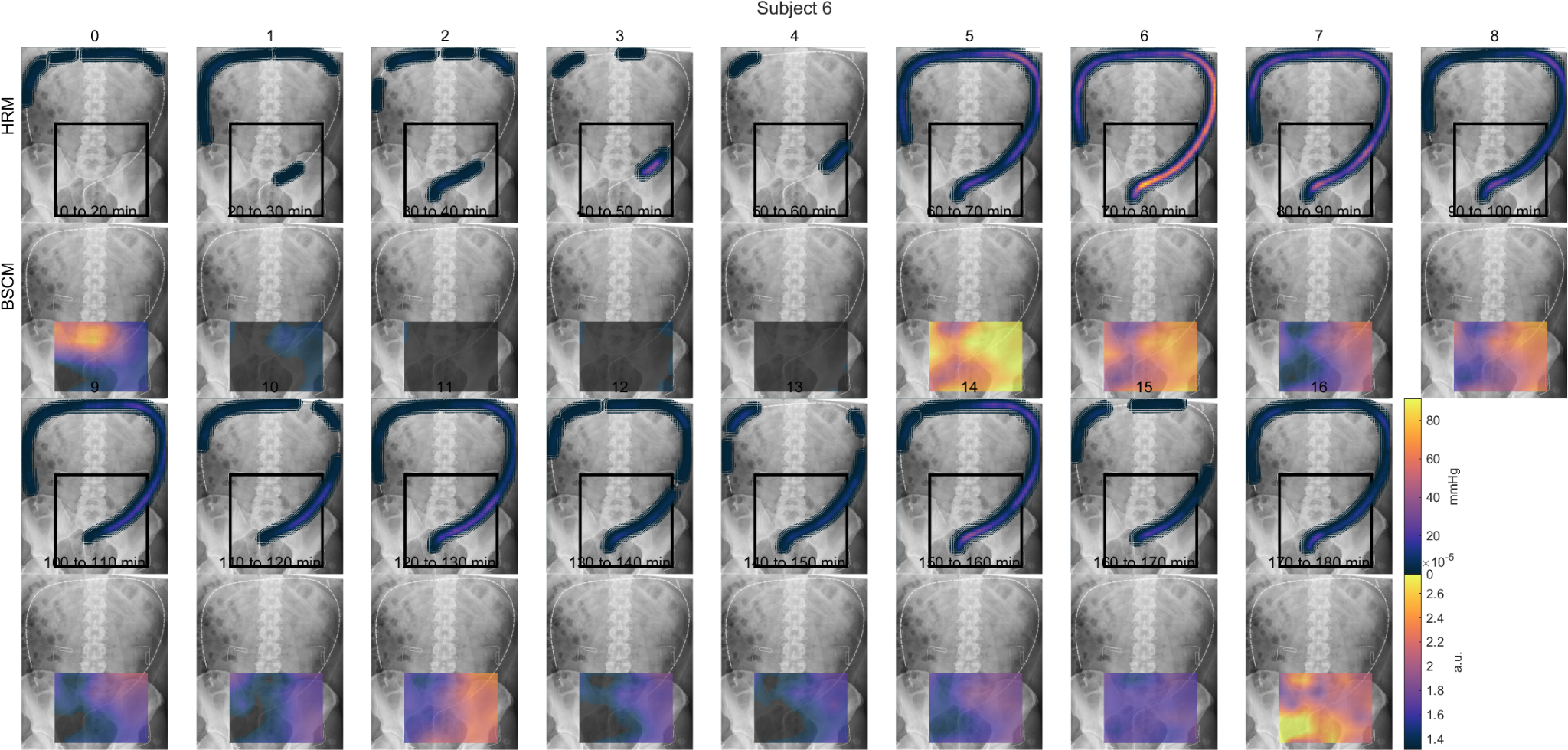


# Figure S3. Analysis of HRCM frequency distributions pre and post-meal.

**
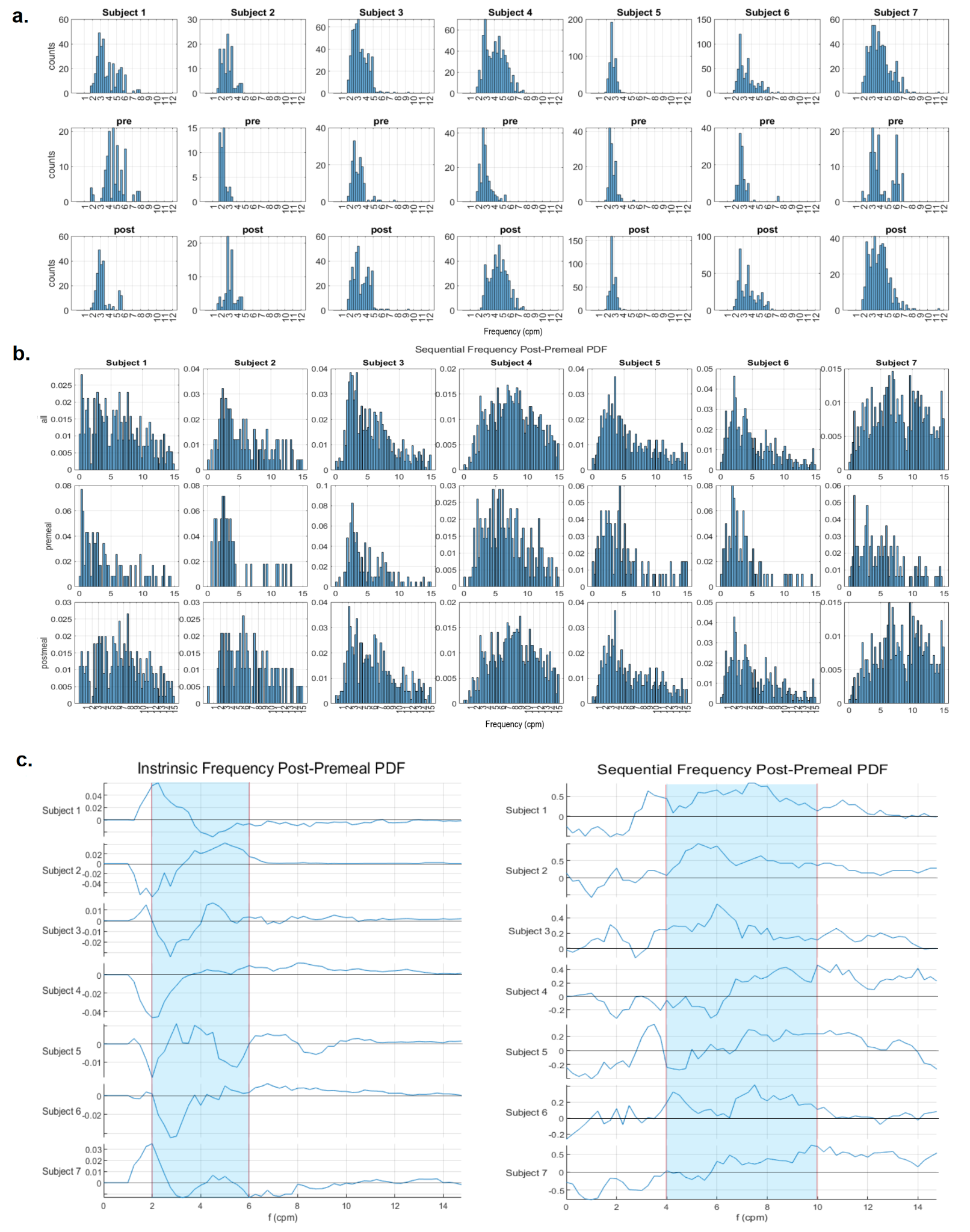
**

**Figure S3: Frequency distributions and changes pre and post-meal.** Histograms panels of intrinsic (**a**) and sequential (**b**) frequency distributions are split into overall (first row) distribution followed by a subanalysis of pre- (second row) and post-meal (third row) frequency distributions. Frequency is in cycles per minute (X = frequency, Y = epoch count). Graphs from panel (**c**) show the differential between pre- and post-meal intrinsic (left) and sequential (right) frequencies for each subject. Biggest rises post-meal are noted in the 2-6 cpm range for the intrinsic frequency and in the 4-10 cpm range for the sequential frequency.
